# Activated Carbon-Based System for the Disposal of Psychoactive Medications

**DOI:** 10.3390/pharmaceutics8040031

**Published:** 2016-11-07

**Authors:** Yang Song, Mahima Manian, William Fowler, Andrew Korey, Ajay Kumar Banga

**Affiliations:** 1 Department of Pharmaceutical Sciences, Mercer University, Atlanta, GA 30341, USA; Yang.Song@live.mercer.edu (Y.S.); Mahima.Manian@live.mercer.edu (M.M.); 2 Verde Technologies, 12701 Whitewater Drive, Minnetonka, MN 55343, USA; wfowler@verdeenvirotech.com (W.F.); akorey@verdeenvirotech.com (A.K.)

**Keywords:** lorazepam, diazepam, buprenorphine, activated carbon, deactivation, psychoactive, adsorption, desorption

## Abstract

The misuse and improper disposal of psychoactive medications is a major safety and environmental concern. Hence, the proper disposal of these medications is critically important. A drug deactivation system which contains activated carbon offers a unique disposal method. In the present study, deactivation efficiency of this system was tested by using three model psychoactive drugs. HPLC validation was performed for each drug to ensure that the analytical method employed was suitable for its intended use. The method was found to be specific, accurate and precise for analyzing the drugs. The extent and rate of deactivation of the drugs was determined at several time points. After 28 days in the presence of activated carbon, the extent of leaching out of the drugs was evaluated. Deactivation started immediately after addition of the medications into the disposal pouches. Within 8 h, around 47%, 70% and 97% of diazepam, lorazepam and buprenorphine were adsorbed by the activated carbon, respectively. By the end of 28 days, over 99% of all drugs were deactivated. The desorption/leaching study showed that less than 1% of the active ingredients leached out from the activated carbon. Thus, this deactivation system can be successfully used for the disposal of psychoactive medications.

## 1. Introduction

Psychoactive drugs are increasingly being prescribed as antidepressants or for the treatment of insomnia and for pain relief. A large share of prescriptions for older adults are for psychoactive, mood-changing drugs that carry the potential for misuse, abuse, or dependency. According to the survey sponsored by the Substance Abuse and Mental Health Services Administration (SAMHSA), the non-medical use of prescription psychotherapeutics surpassed the total illicit use of cocaine, hallucinogens and heroin as the leading drugs of abuse in individuals over 12 years of age [1]. In 2014, approximately 6.5 million illicit drug users in the US had reported nonmedical use of psychoactive drugs including pain relievers, tranquilizers, stimulants and sedatives in their lifetime [2]. Such widespread drug abuse arises from not only the greater prescribing by medical practitioners, but also from misconceptions about their safety. Since their introduction in the late 1950s, benzodiazepines have become the most widely prescribed anxiolytics and hypnotics in medical practice. Both diazepam and lorazepam belong to the benzodiazepine class of drugs and can be used to treat anxiety disorders, induce sleep or reduce agitation in status epilepticus [3]. Diazepam became one of the top selling drugs of all time due to its potency, high bioavailability and onset of action. However, the same factors also resulted in a high risk of dependence and abuse [4]. In case of lorazepam, drug abuse can result in tolerance, dependence and some incompletely reversible effects, and hence the drug is recommended for short term use due to its physical addiction potential [5]. Suboxone^®^ contains buprenorphine and naloxone in a fixed ratio and is widely used for the treatment of opioid addiction [6]. While the use of buprenorphine has been increasing in recent years, buprenorphine is around 30 times stronger than morphine in many of its effects and safety still remains an issue as co-consumption with other psychoactive drugs has been frequently reported [7]. In addition, there are several reports that demonstrate the diversion and misuse of Suboxone^®^ both for self-medication and for producing euphoria [8,9].

Another serious issue of concern is the disposal of leftover unused or expired psychoactive medications. Several studies have reported factors that lead to the accumulation of unwanted medications [10], the approaches to minimize or reduce this accumulation and the factors that encourage drug disposal in sewers in comparison to recommended means like take-back programs [11,12]. Due to such widespread drug abuse and inadequate disposal, it is relatively common to find these drugs and their metabolites in the sewage system. Most consumers would prefer to throw the unused drugs in the normal trash or just flush them down the toilet. Flushing the drugs without proper deactivation can pollute our water system and contaminate food supplies. The detection of pharmaceuticals was first reported in 1976 in the treated waste water in USA [13]. In recent years, many pharmaceutical compounds have been detected in the environment including anti-inflammatory drugs, cancer therapeutics and tranquillizers [14]. Even though the concentration of drugs may be low, it can still hurt marine life and also affect human health. This inadequate disposal of misused/expired medications has even led to their detection in surface water as well as tap water. For this purpose, the US Food and Drug Administration (FDA) has suggested mixing unused drugs with cat litter or coffee grounds which can then be disposed in regular trash. But this is not an effective deactivation process and the drug can still be extracted and has high abuse potential. Thus, mixing psychoactive dosage forms with cat litter or coffee ground cannot effectively prevent drug theft and abuse [15]. The United States Drug Enforcement Administration (DEA), the Food and Drug Administration (FDA) and many other agencies recommend medicine take-back programs as the best way to dispose unused or expired psychoactive medicines. Between 2010 and 2014, even though more than 4 million pounds of medications had been taken back, only about 20% people participated in take-back programs due to lack of awareness about these programs [16]. These programs utilize incineration to dispose medications which could produce toxic air emission including smog inducing gases, ozone depleting agents, and other by-products, finally resulting in air and environmental pollution. Hence, a safer, more convenient and less expensive drug disposal method is critically required.

The drug deactivation system investigated in this study offers a unique disposal method to deactivate unused, residual or expired medications in such cases. This drug deactivation system is based on MAT_12_™ Molecular Adsorption Technology which can deactivate the active pharmaceutical ingredients and dosage forms by adsorbing and firmly binding with activated carbon [17]. For the purpose of this study, the term “deactivation” is used to signify the irreversible adsorption of the psychoactive substances by activated carbon. These activated carbon granules are contained in a water soluble film packet and packaged in a sealable outer pouch. Unused tablets or other dosage forms can be placed in the outer pouch with addition of warm tap water and the pouch can then be sealed. On adding water into the outer pouch, the inner water soluble film dissolves and activated carbon is released to mix with the medications. Once the drug mingles with activated carbon, the efficiency of the deactivation process is dependent on characteristics of the active pharmaceutical ingredient and its dosage form. This study aimed at testing the ability of this drug deactivation system by using psychoactive dosage forms like diazepam and lorazepam tablets and buprenorphine sublingual film in order to determine if this system provides a simple, safe and promising way for consumers to properly dispose their unused, residual or expired medications. Prior to analyzing the drug content in the pouches, validation of the analytical method was performed in order to ensure that this method was relatively simple, accurate and precise for testing the efficiency of this system. A desorption study was also performed to test the possibility of leaching out of adsorbed drug substances from activated carbon in order to simulate the landfill situation where there is a potential for pharmaceutical wastes to seep into groundwater supplies.

## 2. Materials and Methods

### 2.1. Materials

Active pharmaceutical ingredients used in this study were purchased from Sigma-Aldrich (St. Louis, MO, USA), while Suboxone^®^ sublingual films, generic diazepam tablets and generic lorazepam tablets and deactivation pouches were obtained from Verde Environmental Technologies Inc. (Minnetonka, MN, USA). The pouch contains 15 g activated carbon granules which is sealed by a water soluble film. Once warm tap water is added into the pouch, the film dissolves and the released carbon mixes with the medications. Acetonitrile, methanol and potassium phosphate monobasic were purchased from Fisher Scientific (Pittsburgh, PA, USA). Potassium phosphate dibasic and ethanol were purchased from Sigma-Aldrich (St. Louis, MO, USA). Nylon filters (0.22 µm) used for sample filtration were purchased from Medsupply Partners (Atlanta, GA, USA).

### 2.2. Methods

#### 2.2.1. HPLC Validation Study

A validation study was performed for all the drugs in order to ensure that the analytical method employed was suitable. The methods were conducted using an isocratic and gradient reverse phase technique and the validation criteria including linearity, specificity, accuracy and precision of the method were then tested. Fresh stock (1 mg/mL) was prepared with active ingredient and diluted to make standard samples ranging from 0.1 to 50 µg/mL. Three standard samples with concentrations of 1, 2.5 and 25 µg/mL (*n* = 3) that were within the calibration levels were used to test the accuracy and precision. 

All the samples were analyzed by using a Waters Alliance 2795 system (Waters Corporation, Milford, MA, USA) equipped with a Waters 2998 PDA (Waters Corporation, Milford, MA, USA). For analyzing diazepam and buprenorphine, a Kinetex EVO C18 (150 × 4.6 mm^2^, 5 µm, Phenomenex, Torrance, CA, USA) was used. The mobile phase for diazepam consisted of acetonitrile and 20 mM potassium phosphate buffer (pH 2.5, 40:60% *v*/*v*). The flow rate was 1.2 mL/min and UV wavelength was set at 230 nm. For the HPLC analysis of buprenorphine, acetonitrile and 10 mM potassium phosphate buffer adjusted to pH 6 (83:17% *v*/*v*) was used as mobile phase. The flow rate was 1.0 mL/min and wavelength was 212 nm [18]. Lorazepam was detected using an Xbridge BEH Phenyl column (50 × 4.6 mm^2^, 2.5 µm, Waters Corp., Milford, MA, USA). Acetonitrile and water were used as mobile phase delivered under a gradient program. The composition is shown in Table 1. The flow rate was 1.0 mL/min and detection wavelength was 229 nm.

#### 2.2.2. Deactivation of Pharmaceutical Dosage Forms

**Figure 1:**
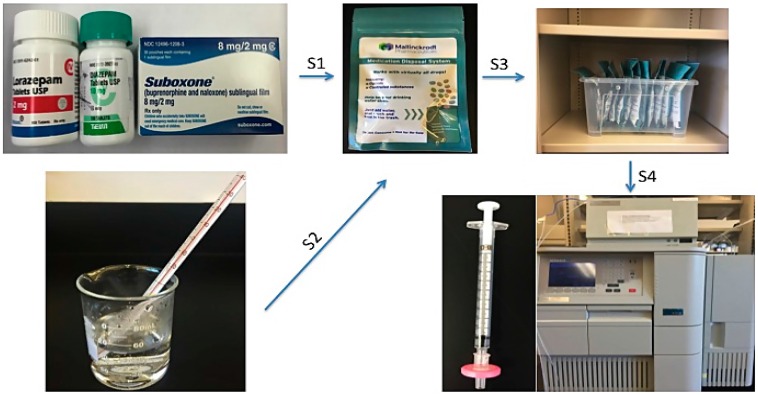
Schematic representation of deactivation study of psychoactive medications. S1: Medications were added to the deactivation pouches; S2: 50 mL warm tap water was added to each pouch; S3: Pouches were stored upright and undisturbed at room temperature; S4: Samples were collected at different time points and analyzed by HPLC.

The deactivation of the different dosage forms was tested using the three model psychoactive medications. Ten diazepam tablets (10 mg), ten Suboxone^®^ sublingual films (8 mg) containing buprenorphine as one of the active ingredients and ten lorazepam tablets (2 mg) were placed into individual pouches separately followed by addition of 50 mL of tap water warm at a temperature of about 43 °C. In order to mix the dosage forms, activated carbon and warm water properly, pouches were shaken for 10 s at the rate of 1 shake per second, followed by a waiting period of 30 s to release the air bubbles from charcoal. After ensuring that all the medications remained at the bottom of the pouch, the pouches were then sealed, stored upright and undisturbed at room temperature. Separate pouches were set up for each time point and samples were collected from pouches (*n* = 2) at 8 h, 1, 2, 4, 7, 14, 21 and 28 days. Figure 1 is a schematic representation of the deactivation procedure followed for dosage forms. Two extra pouches were set up in order to account for any loss during the study. Before taking samples, pouches were shaken mildly from side to side to ensure the medications mixed homogenously in the pouch. Samples were then filtered with 0.22 μm nylon filter and analyzed by the validated HPLC methods. The deactivation rate was calculated as follows

$$\%\;\mathrm{Reacted}=\frac{\mathrm{Initial}\;\mathrm{amount}\;\mathrm{of}\;\mathrm{drug}\;\mathrm{in}\;\mathrm{dosage}\;\mathrm{form}-\mathrm{Amount}\;\mathrm{of}\;\mathrm{drug}\;\mathrm{in}\;50\;\mathrm{mL}\;\mathrm{water}}{\mathrm{Initial}\;\mathrm{amount}\;\mathrm{of}\;\mathrm{drug}\;\mathrm{in}\;\mathrm{dosage}\;\mathrm{form}}\times 100$$

#### 2.2.3. Desorption Study

**Figure 2:**
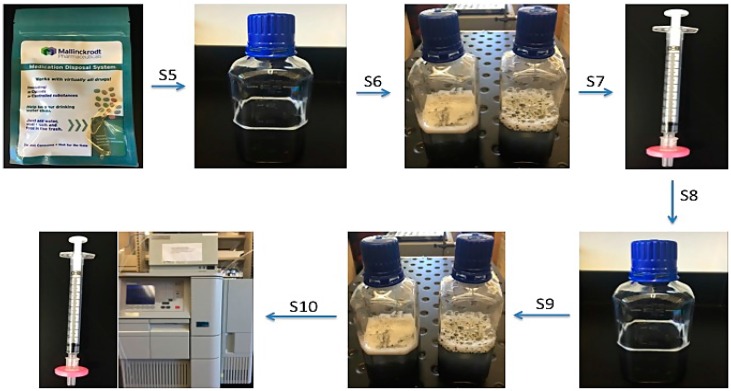
Schematic representation of desorption study of psychoactive medications. S5: On the 28th day, contents of each pouch were transferred into container and distilled water was added; S6: Samples were shaken and then stored upright, undisturbed at room temperature for 23 h; S7: Samples were collected on 29th day; S8: Water was replaced with 30% ethanol; S9: Samples were shaken and stored undisturbed at room temperature for an additional 23 h; S10: Samples were collected on the 30th day and all samples were analyzed by HPLC.

A desorption or washout study was performed following the deactivation study, in order to determine the potential for leaching of the drug from activated carbon. Figure 2 shows the schematic outline of the desorption study performed after 28 days. The entire content of each pouch was transferred into an individual container followed by addition of 200 mL distilled water. The samples were shaken for 1 h at 150 rpm, stored upright for 23 h at room temperature, then filtered and analyzed by HPLC. The water was then replaced with 250 mL 30% ethanol, rocked for an additional hour and stored for 23 h at room temperature. After that the samples were taken from the container, filtered and analyzed by HPLC.

## 3. Results

### 3.1. HPLC Validation

#### 3.1.1. Linearity

**Figure 3:**
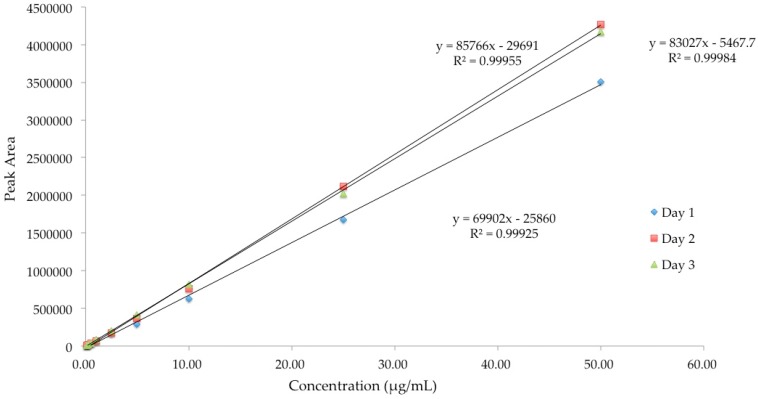
Linearity of HPLC method for analysis of lorazepam.

**Figure 4:**
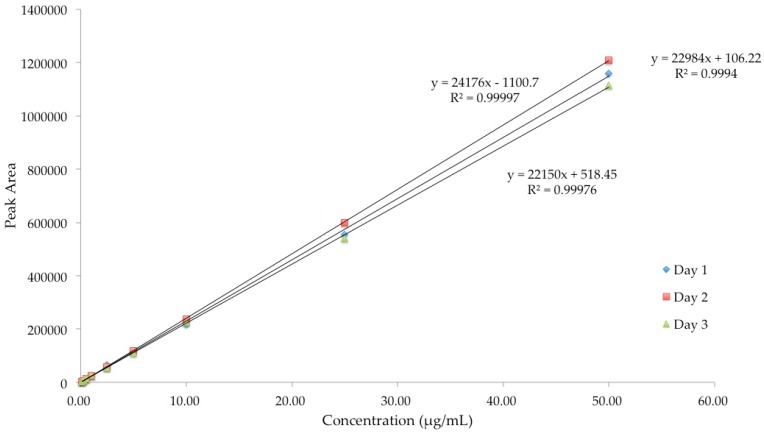
Linearity of HPLC method for analysis of buprenorphine.

**Figure 5:**
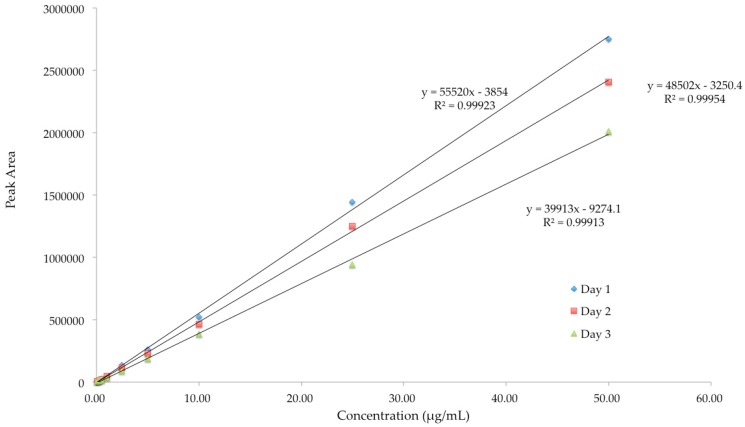
Linearity of HPLC method for analysis of diazepam.

Linearity of an analytical method demonstrates a proportional relationship of peak area and the concentration of analyte in the samples over a definite range. Linearity of the analytical methods for diazepam, lorazepam and buprenorphine was established within the range of 0.1 to 50 μg/mL. Good correlations between peak area and drug concentrations were obtained with *R*^2^ ≥ 0.99 for all the three drugs (Figure 3, Figure 4 and Figure 5).

#### 3.1.2. Specificity

**Figure 6:**
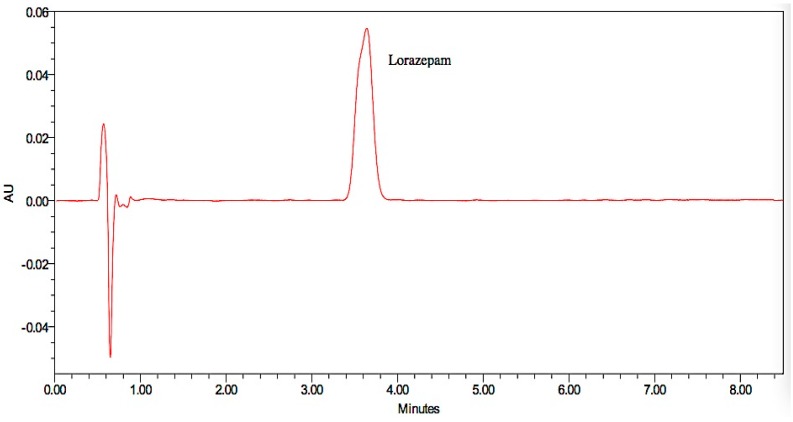
Representative chromatograms of the lorazepam drug sample.

**Figure 7:**
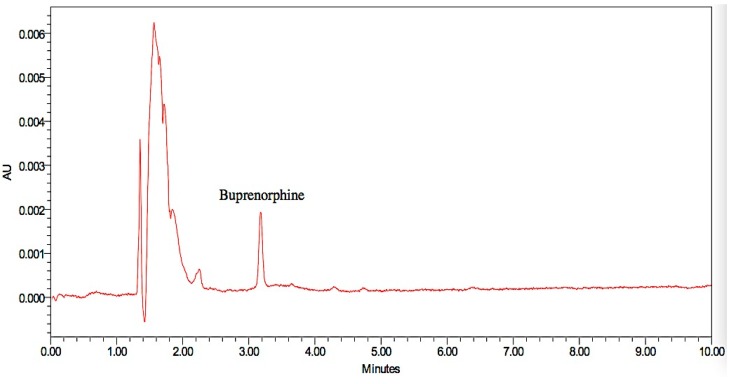
Representative chromatograms of the buprenorphine drug sample.

**Figure 8:**
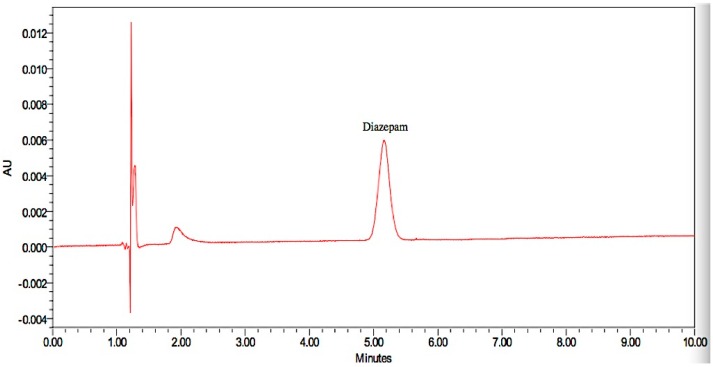
Representative chromatograms of the diazepam drug sample.

Representative chromatograms obtained from the injected drug solutions are presented in Figure 6, Figure 7 and Figure 8. Specificity is used to indicate the ability of analytical methods to detect the analyte of interest. The analytical method should not be affected by the presence of impurities or excipients in the samples [19]. No interference peak from excipients or impurities was observed near the retention time for all drug samples which indicated the specificity of the analytical methods.

#### 3.1.3. Accuracy and Precision

The results of the intra and inter-day accuracy and precision are shown in Table 2 and Table 3. The precision and accuracy of analytical method was determined by calculating the percentage deviation of the calculated concentration and the theoretical concentration [20,21]. The intra-day accuracy of all the three drugs was within the range of 100% ± 10% while the intra-day precision did not exceed 5%. Assay precision was calculated by using the formula Percent coefficient of variation (%CV) = (S.D./Mean measured concentration) × 100 where S.D. is the standard deviation of mean measured concentration. Table 3 shows the results of the inter-day accuracy and precision which were also within an acceptable range. These results show a good capability of response of HPLC system to different concentrations of samples.

### 3.2. Deactivation Study

**Figure 9:**
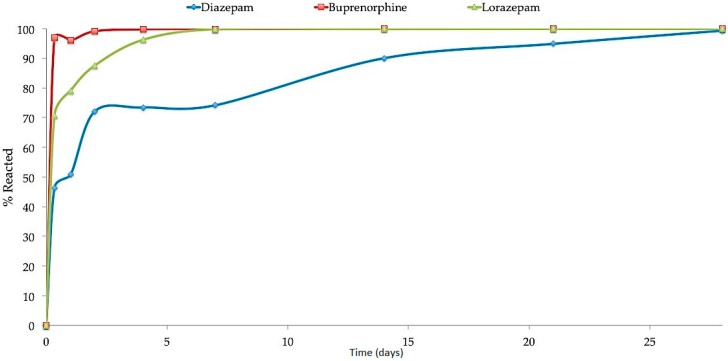
Deactivation profile of diazepam, buprenorphine and lorazepam.

Deactivation of diazepam tablets, lorazepam tablets and buprenorphine containing sublingual films was performed using activated carbon. Each pouch contained 50 g activated carbon granules and either ten tablets of diazepam or lorazepam or ten Suboxone^®^ sublingual films were added. After the addition of warm tap water, about 70% of three medications were adsorbed by the activated carbon within 8 h (Figure 9). In case of diazepam, only around 46% of the drug was adsorbed and more than 50% of diazepam still remained in the water. The amount of lorazepam measured in the pouch at the end of 8 h was 6 mg, which was around 30% of total amount of drug added into the pouch. For diazepam and lorazepam, about 72% and 87.5% drug was deactivated respectively after 2 days. More than 96% of buprenorphine was deactivated by the end of 8 h which increased to 99% after 2 days of adsorption. Medications continued to be adsorbed over time with an average total adsorption of 96.44% by the end of 14 days. After 28 days, adsorption by the activated charcoal resulted in more than 99% deactivation/adsorption of diazepam, lorazepam and buprenorphine. Low levels of residual drug were observed in the pouch, which was less than 1% for all three psychoactive medications.

### 3.3. Desorption Study

**Figure 10:**
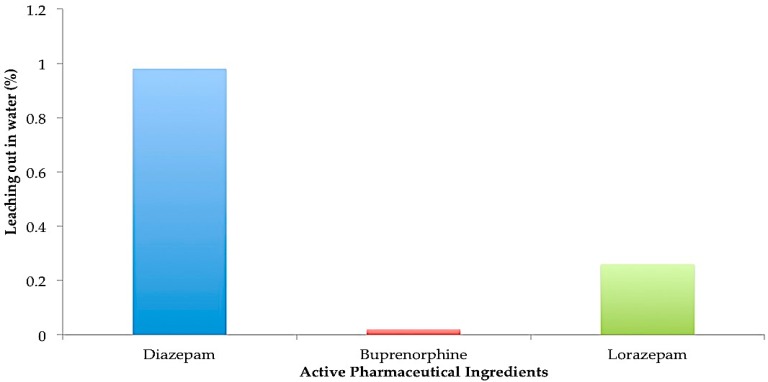
Desorption study in water.

**Figure 11:**
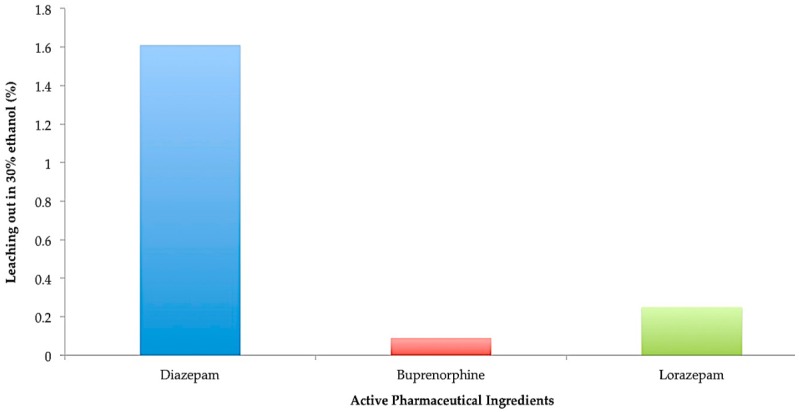
Desorption study in 30% ethanol.

The desorption or washout study was carried out in order to simulate a landfill situation and to test for potential desorption of the various drugs from activated carbon. For the washout phase, contents of pouches after 28 days of the adsorption study were emptied into 500 mL container and excess water was added to make up volume to 250 mL. Addition of excess water facilitated complete release of active pharmaceutical ingredient from tablets or films with continuous adsorption by the activated carbon. The percentage of diazepam, lorazepam and buprenorphine that leached out after 1 day of desorption is shown in Figure 10. The water was then replaced with 30% ethanol to determine the leaching potential from the adsorbent. In this case, about 1.6% of diazepam leached out from the activated carbon. For buprenorphine and lorazepam, only 0.11% and 0.25% of drug leached out from the activated charcoal after 24 h respectively as seen in Figure 11.

## 4. Discussion

The abuse of psychoactive drugs constitutes a growing problem and it is estimated that by 2020, about 2.7 million adults will have used prescription drugs due to dependency or for recreational purposes [22]. In addition, the increased prescribing of psychoactive drugs has raised concerns about proper drug disposal once they are no longer required by patients. Traditional/improper disposal such as flushing medicines down the toilet or pouring them down the drain can increase the harm of accidental exposure especially in children as well as lead to environmental contamination. Scientists have not only detected the presence of these medications in water bodies which can affect marine life but also in sewage water which can potentially affect animal and human life. Methods which have been recommended by FDA or U.S. Drug Enforcement Administration (DEA) to deal with the disposal of these unused drugs such as mixing with ground coffee or cat litter cannot effectively deactivate the drugs [23,24]. Take back programs are regarded as the safest and most environmentally protective method for drug disposal but the problem is the scarcity of collection sites, general unawareness in the public and complications due to transfer of controlled substances [25]. After returning the unwanted/expired medications to drop-off locations, these medications are then securely transferred for further incineration. Even though high temperature incineration meets industry standards for safe disposal, it can still cause increased air emissions of pollutants and ozone depleting agents resulting in environmental pollution.

Hence, the drug deactivation system investigated in this study is an excellent alternative and offers a simple and convenient method to safely deactivate and dispose unused or expired psychoactive medications. Activated carbon is obtained by thermal decomposition of carbon based materials such as coal, coconut or wood. The purpose of this activation procedure is to achieve a high internal surface area which is good for the adsorption of the drug from the formulation to the activated carbon. This large surface area is due to the presence of small, low volume pores on the charcoal [26] where the pore size distribution contributes to the efficiency of the activated carbon in the drug adsorption. Activated carbon has numerous micropores in comparison to charcoal which provides maximum bonding surface area for drug binding. This granular activated carbon is already being used in water treatment processes for removal of micropollutants including pharmaceuticals and endocrine disruptors [27].

This study was performed as per the protocol and guidelines established by Verde Technologies Inc. in association with the National Institute on Drug Abuse (NIDA) which involved the use of duplicate pouches in order to evaluate the deactivation rate of the model psychoactive medications. In addition, as separate pouches were used for individual time points, performing the study in duplicate helped to minimize the handling and use of these controlled substances. Each pouch contained ten dosage forms of psychoactive substances which were found to be adequate to analyze the deactivation and desorption of each dosage form. A previously published study performed in our laboratory had compared the efficacy of various deactivation agents with activated carbon for deactivation of various active pharmaceutical ingredients. Adsorption by activated carbon resulted in a faster deactivation rate for all the drugs used in the study [23]. The Molecular Adsorption Technology (MAT_12_™) used in the disposal system neutralizes or adsorbs the psychoactive ingredients from different dosage forms. Thus, the activated carbon in the disposal system will bind to the different pharmaceutical formulations, successfully deactivate the psychoactive drugs and can then be safely disposed in regular trash. 

The adsorption capacity of activated carbon is also related to the molecular weight of the adsorbents. With increasing molecular weight, the adsorbability of a compound will also increase. In this study, the molecular weight of all the active compounds was less than 500 kDa. However, as the molecular weight of buprenorphine was higher than that of diazepam and lorazepam, the adsorption of buprenorphine was much faster in comparison to the other two drugs. More than 99% of buprenorphine was adsorbed by activated carbon by the end of the second day. Clinically, Suboxone^®^ sublingual films can dissolve in 6–7 min [28] which may also contribute to its faster adsorption as compared to lorazepam and diazepam immediate release tablets which may show a slower dissolution rate. In addition, the non-polar surface of activated carbon preferentially adsorbs hydrophobic compounds which could also potentially explain the faster deactivation rate of buprenorphine (more than 90%) due to its increased hydrophobicity in comparison to the benzodiazepine drugs [29]. 

Mixing drugs with the currently recommended cat litter or coffee grounds may still result in availability of drugs to groundwater when placed in landfills. These emerging contaminants from landfill leachate can then contaminate groundwater for decades. Hence, the robustness of the disposal system in holding on the adsorbed psychoactive drugs was tested by exposure to stress conditions by agitating the pouches and thus simulating a landfill situation. The results of the washout study showed that more than 99% of the drug was deactivated in the presence of water. On replacing with ethanol, less than 1% of adsorbed drug leached out from the activated carbon which suggests that as the discarded drug product would be almost fully deactivated and insoluble in water, this should ultimately contribute to a much lower exposure of groundwater to such pharmaceuticals.

## 5. Conclusions

The effectiveness of the activated carbon based drug disposal system was examined using three model psychoactive medications. The deactivation system successfully adsorbed and deactivated about 70% of the psychoactive medications by 8 h and more than 99% within 28 days and did not release adsorbed drug substances when exposed to large volumes of water or 30% ethanol. Thus, this unique system is simple, safe and user-friendly for patients who can deactivate unused or expired psychoactive medications from the comfort of their homes.

## Figures and Tables

**Table 1:** HPLC gradient method for Lorazepam.

Time (min)	Flow rate (mL/min)	Acetonitrile	Water
0	1.0	5%	95%
7.2	1.0	60%	40%
7.3	1.0	5%	95%
8.5	1.0	5%	95%

**Table 2:** Intra-day accuracy and precision of HPLC methods.

Drug	Concentration (µg/mL)	Mean measured concentration (µg/mL ± SD)	Precision (%CV)	Accuracy (%)
Diazepam	1	0.92 ± 0.042	4.6	92.00
	2.5	2.47 ± 0.041	1.66	98.66
	25	23.96 ± 0.282	1.18	95.82
Buprenorphine	1	1.19 ± 0.048	4.06	92.00
	2.5	2.42 ± 0.134	5.57	96.72
	25	25.18 ± 0.776	3.08	100.70
Lorazepam	1	1.11 ± 0.023	2.11	92.00
	2.5	2.42 ± 0.083	3.43	97.00
	25	24.14 ± 1.132	4.69	96.58

**Table 3:** Inter-day accuracy and precision of HPLC methods.

Drug	Concentration (µg/mL)	Mean measured concentration (µg/mL ± SD)	Precision (%CV)	Accuracy (%)
Diazepam	1	1.07 ± 0.091	8.46	106.73
	2.5	2.49 ± 0.05	2.17	99.60
	25	24.81 ± 1.111	4.48	99.26
Buprenorphine	1	1.12 ± 0.082	7.29	112.00
	2.5	2.53 ± 0.206	8.15	101.36
	25	24.84 ± 1.21	4.87	99.35
Lorazepam	1	1.12 ± 0.024	2.11	92.00
	2.5	2.47 ± 0.133	5.37	98.96
	25	24.49 ± 1.151	4.7	97.96
